# Precision of Fungal Resistance Test Method for Cereal Husk-Reinforced Composite Construction Profiles Considering Mycelium Removal Techniques

**DOI:** 10.3390/ma18020411

**Published:** 2025-01-17

**Authors:** Ewa Sudoł, Ewa Szewczak, Mariia Goron, Ewelina Kozikowska

**Affiliations:** 1 Construction Materials Engineering Department, Instytut Techniki Budowlanej, 00-611 Warsaw, Poland; m.goron@itb.pl (M.G.); e.kozikowska@itb.pl (E.K.); 2 Group of Testing Laboratories, Instytut Techniki Budowlanej, 00-611 Warsaw, Poland; e.szewczak@itb.pl

**Keywords:** repeatability, reproducibility, polymer composites, fungi, flexural properties

## Abstract

Many test methods used in the assessment of construction products are characterised by poor precision, which is reflected in the discrepancies of results obtained by different laboratories. The test procedure for fungal resistance of natural particle-reinforced composite construction profiles has not been fully specified, which may lead to such discrepancies and erroneous utility assessment. In this study, the precision of the method and the influence of the mycelium removal on the fungal resistance were assessed based on the flexural strength and modulus of elasticity test results obtained for millet- and oat husk-reinforced PVC composites exposed to *Coniophora puteana*. The study revealed low precision of the test method, the coefficient of variation, in which, based on the standard deviation of interlaboratory reproducibility for one of the tested composites, was even higher than 20%. Additionally, it was found that the method of mycelium removal can significantly (at the level of 16% difference between flexural strength results) affect the resistance test results. This indicates the need to modify the test method and clearly specify the recommended method of cleaning samples after exposure to fungi.

## 1. Introduction

The development of new materials and products in the construction sector requires the development of new test methods. However, not all test methods, both those used so far and those developed recently, provide reliable, credible results. The standardisation of test methods is intended to provide a uniform system for assessing products, including construction products, and enable the comparison of their performance properties. The basic metrological condition that must be met to achieve this goal is the comparability of the results. According to VIM 3 [1], the measurement results are metrologically comparable when they are both metrologically traceable to the same measurement unit, such as the meter. It is relatively simple to compare the measurement results, but it should be noted that laboratory test results cannot always be treated in the same way as the measurement results. The already withdrawn document EA-4/16 [2] aptly presented the substance of the difference between the terms measurement and test. First, it distinguished the results of these activities into a ‘measurand’ in the case of measurement and a ‘characteristic’ in the case of the test. In practice, a measurand is a physical quantity that has a ‘true value’, independent of the test method. Typical examples of measurement are linear dimensions, mass, and temperature. In the case of testing, the result is determined by the test method itself, and there is no well-defined ‘true value’ that can be obtained using another test method. Tests (reaction to fire, fire resistance, impact resistance, resistance to climatic conditions, water absorption, adhesion, etc.) are predominant in most activities aimed at assessing building materials and elements. In the case of tests, even if the results are expressed numerically and are comparable owing to the same measurement unit, they cannot always be compared with each other as they may depend on the test method used [3].

As research [4,5,6,7] indicates, even the standardisation of test methods may not ensure uniform product evaluations. Uncertainty plays a major role when assessing the compliance of the test results with the criteria [8]. However, the uncertainty of measurement, which is estimated by the laboratory based on the results of calibrations of its equipment and possible in-laboratory experiments, most often does not include a component related to the reproducibility of the test method. In many cases, the level of reproducibility of the methods developed by standard committees is unsatisfactory and does not allow a reasonable assessment of compliance with the criteria, especially in the case of multi-stage methods [5,9]. Often, this is owing to the underspecification of the test procedures or their stages, which, in the sense of the paradigm presented by Walker [10], represents uncertainty in the structure of the test model.

One of the most important tests for building materials and elements is their resistance to environmental factors. With regard to natural fibre-reinforced composites, as well as other composites reinforced with natural particles, such as cereal husk-based composites, the level of susceptibility to environmental factors is of crucial interest [11,12]. Studies on the resistance to environmental factors have paid a lot of attention to the change in properties as a result of artificial ageing involving UV light [13,14,15], water, high temperature and hydro-thermal cycles involving water, low and high temperature [14,16,17,18], and microorganisms [19,20,21,22,23,24,25,26,27,28,29,30,31]. It was found that artificial ageing has the most significant effect on bending properties, followed by fungi, and hydro-thermal cycles the least [12]. The hydrophilic nature of the plant filler means that, in contact with water, it swells easily, causing cracks to form in the hydrophobic polymer matrix. The interaction between the lignocellulosic particles and the polymer also deteriorates, reducing the stress transfer capacity between the fibres and the matrix and resulting in a deterioration of the composite performance [11,17]. Composites are susceptible to biotic agents, particularly fungi of the class *Basidiomycetes* [18,19], classified in modern taxonomy as the subfamily *Agaricomycotina*. The polymer matrix is characterised by rather low susceptibility to biodegradation processes. The fungal resistance of a composite depends mainly on the type, amount, and size of the filler and its degree of dispersion in the matrix [17,18,24]. The development of microorganisms is made possible by the nutrients present in plant particles, lignin, and cellulose. Composites reinforced with low-absorbing fibres are less susceptible to fungi than those using high-absorbing fibres [23]. It has been established that the fibres in composites reach up to 70% moisture content, which, with the right temperature and ph, provides optimal conditions for the growth of microorganisms [22]. White decay fungi, such as *Coriolus versicolor*, primarily attack lignin, while brown decay fungi, such as *Coniophora puteana*, primarily degrade cellulose [26]. Proper fibre dispersion in the matrix is an ally of bioresistance. It promotes good interfacial adhesion by reducing voids, which act as channels for fungal movement through the material, facilitating enzyme transport [20]. The resistance to microorganisms decreases as the amount of filler increases [31]. Previous studies have recorded varying susceptibility to particular strains of fungi. The most intensive growth was observed for *Trametes versicolor* and *Coniophora puteana*, with weight loss not exceeding 5% and no significant change in flexural modulus [22,23,24]. Nevertheless, there are known cases of decreases in flexural strength of up to 30%, modulus of elasticity of up to 40% [24,29,31], and impact strength of up to 16% [30].

Tests of resistance to biotic factors comprise several stages, such as specimen preparation, exposure to microorganisms, and post-exposure property tests, including changes in morphological, physical, or mechanical properties and comparison with the initial state.

Several sources of uncertainty that affect the final result can be identified in each step.

Differences in microbial activity are important in tests of resistance to biotic factors. This impact can be reduced by controlling the strain quality and culture conditions prior to test sample exposure [19,20]. The results can also be influenced by the method of mycelium removal as well as the methods used to assess the performance properties. During long-term exposure to fungi, mycelia tend to overgrow in the samples. The mycelium can be harmful to human health and interfere with the performance of the test equipment; therefore, it should be removed after completing the incubation and before further testing. This stage of the procedure is often overlooked in descriptions of test methods, including test standards [32]. Some authors have proposed a cleaning method. The samples can be cleaned with a soft brush [30], pressurised water [31], or sandblasting [22]. The test procedure for natural fibre composite fungal resistance [33] has also not been fully defined in this area, which may lead to discrepancies in the results obtained by different laboratories.

As indicated by the results of previous evaluations of metrological properties of some test methods [5], their precision seems to be insufficient for the purposes of uniform evaluation of construction products. In this study, the precision of the resistance to biotic factors test method was analysed. There is no similar analysis in the literature.

The study consisted of exposing samples of natural fibre-reinforced composites to the action of microorganisms and then subjecting them to flexural properties tests. The changes in flexural strength and modulus of elasticity occurring under the influence of fungi exposure were assessed. Two methods were used to remove mycelium from samples after exposures, which is also a novelty of this work. Based on the results of these tests, in the first stage, the precision of the fungus resistance test method was assessed, independently for both methods of mycelium removal. The precision analysis was based on the repeatability variance and intra-laboratory reproducibility variance (tests performed by two independent researchers). Then, the influence of the mycelium removal technique on the results of fungi resistance tests of cereal husk-reinforced polymer construction profiles was analysed, which has not been previously studied. The results of the statistical analysis of the significance of differences in flexural properties after using different mycelium removal methods were also supported by the analysis of the microstructure observed using a scanning electron microscope (SEM). The problem was studied in the case of millet- and oat husk-reinforced PVC composites exposed to *Coniophora puteana*. The results obtained allow for the assessment of the level of precision of the method and the influence of the sample cleaning step, which were not included in the standard test procedure, due to possible discrepancies in the results.

## 2. Materials and Methods

### 2.1. Samples

**Figure 1:**
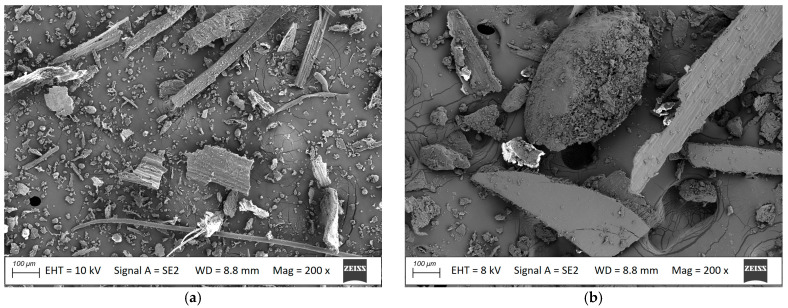
Pulverised (**a**) oat husks and (**b**) millet husks, SEM images, magnification 200×.

The profiles for outdoor terrace flooring were also tested. Polyvinyl chloride (PVC) containing chalk (CaCO_3_) was used as the matrix, and pulverised oats and millet husks were used as fillers. The oat husk filler was dominated by slender particles, up to 50 μm wide and 100 μm or more long (Figure 1a), which can be considered fibres. Pulverised millet husks took on a plate-like shape, with a width of up to 100 μm (Figure 1b). No millet-based fibres were noted.

The compositions of composites are listed in Table 1.

**Figure 2:**
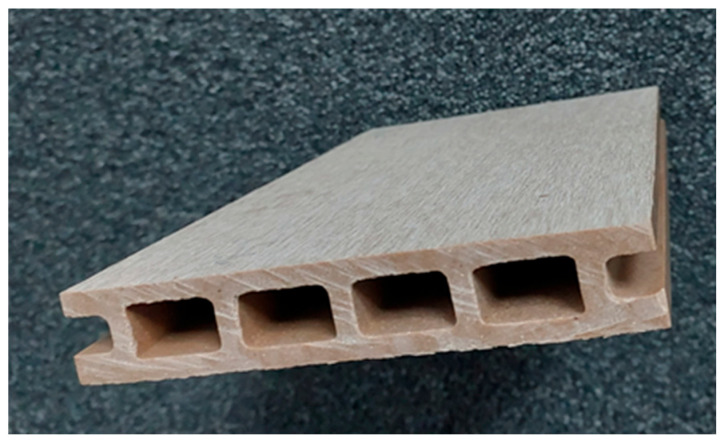
Appearance of profiles from which samples were taken for testing.

The profiles were manufactured under industrial conditions by using an extrusion method. They had a chamber shape, width of 125 mm, height of 22 mm, and a wall thickness of 5 mm. Their face surfaces were flat without milling, but the factory was treated with a metal brush to give them a slightly rough wood-like texture (Figure 2).

Test specimens were cut from the face surface to preserve the original wall thickness. They were obtained from the central part of the chamber, parallel to the ribs. They had dimensions of 80 mm × 10 mm × 5 mm. From both the millet husk-reinforced PVC composite (M) and the oat husk-reinforced PVC composite (O), 46 samples were prepared. Of these, six samples were seasoned under laboratory conditions, and the remaining samples were aged in water for 2 weeks [32]. Distilled water was used, which was changed nine times.

### 2.2. Exposition to Fungi

Lyophilised strain of the fungus *Coniophora puteana* Schumach. BAM Ebw. Fifteen were used to prepare the parental cultures. According to the recommendations of the supplier, the fungus was rehydrated with 1 mL of sterile distilled water. After rehydration, which lasted for 2 h under normal conditions, the fungus was transplanted onto a sterile malt extract agar medium to grow the parental cultures. A medium consisting of 40 g malt extract, 35 g agar, and 1000 mL of water was used. The medium was sterilised in an autoclave at 121 ± 2 °C and a pressure of 1 bar. The fungus was cultured at 22 ± 1 °C and 70 ± 5% humidity for 2 weeks and then transplanted onto freshly prepared malt extract agar medium. After cultivation for one week under the same conditions, subcultures were obtained.

Kolle flasks were prepared to the specification of the test samples and fungi. Fifty mL of malt extract agar medium was poured into each flask, sterilised, and left to solidify horizontally. Subcultures of *Coniophora puteana* with well-developed mycelia were used. Each flask was inoculated with two pieces of mycelia from subcultures under sterile conditions. The flasks were left in an incubation chamber at 22 ± 1 °C and 70 ± 5% until the surface of the medium was completely grown using mycelium. The activity of the fungi was verified in a concurrent test on the sapwood of *Pinus sylvestris*, resulting in a mass loss of 45% after 16 weeks.

Samples previously aged in water were placed on the mycelium with a usable face surface in direct contact. A total of 40 samples of millet husk-reinforced PVC composite and 40 samples of oat husk-reinforced PVC composite were exposed to *Coniophora puteana*. Flasks with samples were held in an incubation chamber at 22 ± 1 °C and 70 ± 5% for 16 weeks. The degree of mycelial growth on the samples was assessed visually according to the following scale: grade 0—no mycelium, grade 1—scarcely developed mycelium, grade 2—medium-developed mycelium, grade 3—well-developed mycelium, and grade 4—very thick mycelium. After, the mycelium was removed.

**Figure 3:**
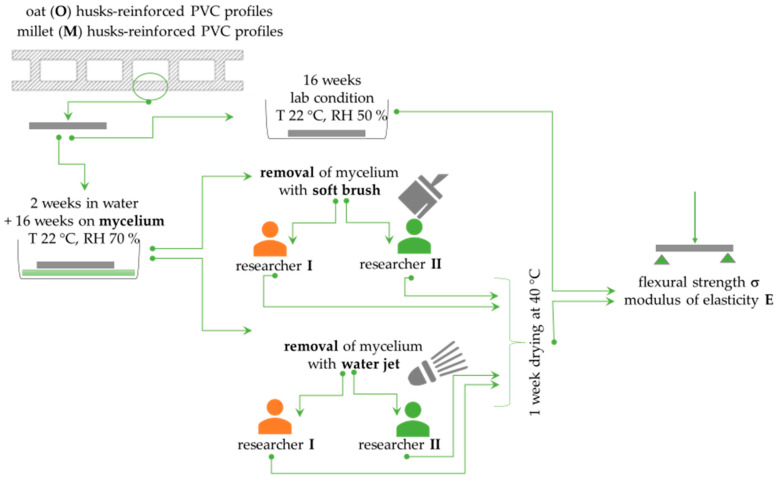
Experimental flowchart.

Mycelium removal was performed independently by two researchers with several years of experience in microbiological testing of building products. Each used two cleaning methods. Half of the samples from the given composite were manually cleaned using a brush. A soft nylon bristle brush with a diameter of 0.18–0.20 mm was used. The brush head measured 30 × 15 mm with four rows of fibre bundles arranged in 10 columns. The other half was cleaned using a water jet. A pressure washer (Kärcher, Poland, Kraków) was used. Water at 23 ± 2 °C was distributed using a lance at 12 bar pressure, at an intensity of 9.2 L/min. No detergent was used for either method. Cleaning was performed carefully until the mycelium was completely removed and visually controlled. After cleaning, all samples were dried for one week at 40 ± 2 °C in a forced-air laboratory dryer. Figure 3 shows the flowchart of the experimental steps.

### 2.3. Microstructure Analysis

The microstructure was examined with Sigma 500 VP cold-field emission scanning electron microscope (Carl Zeiss Microscopy GmbH, Köln, Germany). The tests were carried out at the accelerating voltage of 5 KeV, 8 KeV, or 10 KeV inductive electron beam, using an SE detector. The observation was carried out on samples in initial state and after exposure to *Coniophora puteana*, both unpurified and after removal of the mycelium. All samples were dried for 7 days at T 40 ± 2 °C in a chamber with forced-air circulation before observation, and then a layer of gold was sputtered. SEM observations of the mycelium were performed at 500× and 5000× magnification, while the surfaces of the composites in the initial state and after the fungus culture were examined at 100× magnification. Microscopic observations were carried out on the usable surface of the profiles. Brittle fracture after fungus cultivation was also analysed.

### 2.4. Flexural Properties Test

The flexural strength σ and modulus of elasticity E, were determined, according to ISO 178 [34], using a class 1 strength testing machine (Instron, Darmstadt, Germany). Three-point bending, similar to that in [14,35], was performed. Supports with a 5 mm radius were used, spaced every 64 mm, and a 5 mm radius pressing element was placed in the middle of the span. The samples were then provided with free support. The load was applied to the ground surface at a constant rate of 2 mm/min until it was destroyed. The flexural strength, σ, was calculated according to Equation (1) [34] and expressed in MPa.

(1) $$\sigma =\frac{3FL}{2bh^{2}}$$

where the following are defined: *F*—maximum force, in N; *L*—support spacing, in mm; *b*—sample’s width, in mm; and *h*—sample thickness, in mm.

A load–deflection curve was recorded during bending in a linearly elastic range, including the force and deflection values corresponding to strains ε*_f_*_1_ = 0.0005 and ε*_f_*_2_ = 0.0025. The deflection values of *f*_1_ and *f*_2_ were calculated using Equation (2) [34].

(2) $$f_{1}=\frac{\varepsilon_{f1}L^{2}}{6h};\;f_{2}=\frac{\varepsilon_{f2}L^{2}}{6h}$$

where the following are defined: *L*—spacing of supports, in mm; and *h*—sample thickness, in mm.

The force values recorded when ε*_f_*_1_ and ε*_f_*_1_ strains occurred were used to determine the values of the σ*_f_*_1_ and σ*_f_*_2_ normal stresses. The *E* modulus was calculated according to Equation (3) [34] and is expressed in MPa.

(3) $$E=\frac{\sigma_{f2}-\sigma_{f1}}{\varepsilon_{f2}-\varepsilon_{f1}}$$

where the following are defined: σ*_f_*_1_, σ*_f_*_2_—maximum normal stress corresponding to *f*_1_ and *f*_2_ stress, determined according to (2).

The series of both the millet husk-reinforced PVC composite and the oat husk-reinforced PVC composite are described in Table 2. The initial results were sets of six results obtained by one researcher, and the sets of results after exposure and mycelium removal consisted of ten results for each of the two researchers.

### 2.5. Statistical Analysis Methods

The precision analysis of the method was based on the estimates of the values of the repeatability variance, between-researcher variance, and reproducibility variance obtained for all properties Y: σ_b,_ σ_w,_ E_b,_ E_w_ (Table 2) according to the methodology presented in ISO 5725-2 [36]. The assessment of precision should be preceded by the detection of outliers, and the method recommended by ISO 5725-2 is the Grubbs test, which is based on the assumption of normality of the result distributions. Therefore, first of all, all series of results described in Table 2 were subjected to Shapiro–Wilk tests for normality, which is characterised by a relatively high power. Then, to detect a single deviating value in a dataset, a Grubbs test was performed for all result series.

- The repeatability variance, s_r_^2^, is calculated for all properties Y according to Equation (4).

(4) $$s_{rY}^{2}=\frac{s_{rYI}^{2}+s_{rYII}^{2}}{2}$$

where

$s_{rYI}$—the sample standard deviation of the first operator’s results for quantity Y;

$s_{rYII}$—the sample standard deviation of the second operator’s results for quantity Y.

For the initial result series σ_i,_ and E_i_, the standard deviation of the repeatability was assumed to be the standard deviation of the sample of six results

- The between-researcher variance, s_L_^2^, is calculated for all properties Y according to Equation (5).

(5) $$s_{LY}^{2}=\frac{s_{dY}^{2}-s_{rY}^{2}}{{\overset{̿}{n}}_{Y}}$$

where

s_LY_—between-researcher standard deviation for quantity Y

and

(6) $$s_{dY}^{2}=n_{YI}{\left(\bar{Y_{I}}-\overset{̿}{Y}\right)}^{2}+n_{YII}{\left({\bar{Y}}_{II}-\overset{̿}{Y}\right)}^{2}$$

where

$n_{YI}$, $n_{YII}$—number of samples for quantity Y and operator I and II, respectively;

${\bar{Y}}_{I}$, ${\bar{Y}}_{II}$—average result for quantity Y and operator I and II, respectively;

$\overset{̿}{Y}$—general mean calculated according to Equation (7).

(7) $$\overset{̿}{Y}=\frac{n_{YI}\;\bar{Y_{I}}+n_{YII}\;\bar{Y_{II}}}{n_{YI}+n_{YII}\;}$$

and

(8) $${\overset{̿}{n}}_{Y}={(n}_{YI}+n_{YII})-\frac{\left({n_{YI}}^{2}+{n_{YII}}^{2}\right)}{\left(n_{YI}+n_{YII}\;\right)}$$

- The within-laboratory reproducibility variance, s_R_^2^, is calculated for all properties Y according to Equation (9).

(9) $$s_{RY}^{2}=s_{rY}^{2}+s_{LY}^{2}$$

where

s_RY_: within-laboratory reproducibility standard deviation for quantity Y.

All Equations (4)–(9) are derived from the equations given in standard ISO 5725-2 [36].

To ensure the comparability of the variability of the results for different test result values, all standard deviations were converted to coefficients of variation $v_{Y}$ according to Equations (10) or (11):

(10) $$v_{Y}=\frac{100s_{Y}}{\overset{̿}{Y}},\;\%$$

or for initial tests

(11) $$v_{Yi}=\frac{100s_{Yi}}{\bar{Y}},\;\%$$

The relatively large dispersion of test results (shown in Section 3.3.1 of this article) requires statistical evaluation of whether the difference in means for given result groups (in the case of the described experiment of result sets for the same operator and the same method of sample cleaning) is significant. A typical test used to analyse the statistical significance of differences between result groups is the analysis of variance (ANOVA), which is based on the comparison of variance within result groups and intergroup variance related to the differences between means. One-way ANOVA was used to analyse whether the differences in test results resulting from different surface cleaning methods are statistically significant.

The F-test was used to draw some conclusions regarding differences in intra-group variances of the obtained results. (The test concerns two groups of results from normal distributions. For a larger number of groups, other tests are used, e.g., Bartlett’s).

## 3. Results and Discussion

### 3.1. Surface Assessment

**Figure 4:**
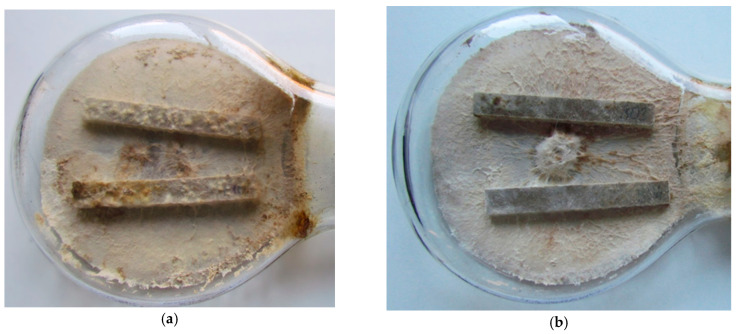
Appearance of specimens after exposure to *Coniophora puteana*: (**a**) millet husk-reinforced composite and (**b**) oat husk-reinforced composite.

After 16 weeks of exposure to *Coniophora puteana*, the degree of mycelial growth on the samples was assessed visually. For the millet husk-reinforced composite, well-developed mycelium, corresponding to grade 3, was observed (Figure 4a). For oat husk-reinforced composite, medium mycelium coverage was noted, which corresponds to grade 2 (Figure 4b). The millet husk-reinforced composite showed more fungal overgrowth, which indicates that the oat husk-reinforced composite is more resistant to fungi [12,13].

**Figure 5:**
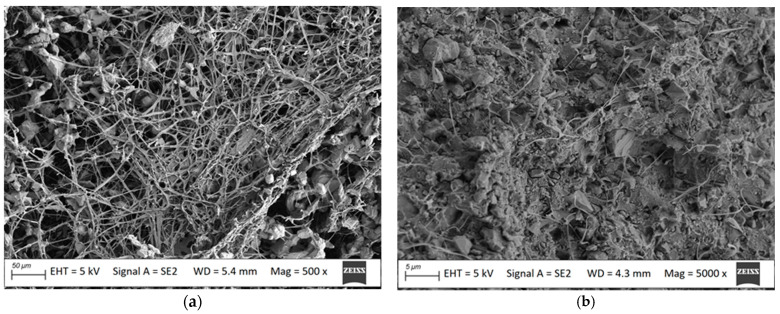
*Coniophora puteana* mycelium (**a**) on the surface before cleaning and (**b**) in cross-section of the oat husk-reinforced composite, magnification, respectively, 500× and 5000×.

The surface of samples before mycelium removal was subjected to SEM analysis. It showed a developed mycelium with distinct spores (Figure 5a) characteristic for the *Coniophora puteana* strain. Mycelium images on the millet husk-reinforced composite were similar to those observed on the oat husk-reinforced composite. Due to the degree of mycelium development completely obscuring the composite, it was not possible to assess the surface morphology of the samples before cleaning. A brittle fracture test was also conducted to see if the mycelium enters inside the composite structure. The microstructure analysis showed that *Coniophora puteana* penetrates inside the walls of the profiles. In the images (Figure 5b), it is visible in the form of a dense network of thin threads, indicating that the fungus was present throughout the composite.

Cleaning of the samples with both a brush and wet jet resulted in complete removal of the mycelium. There were no differences in the surface appearance of the samples owing to the cleaning method. However, there was a significant difference in the surface appearance of the samples with millet husks compared to those with oat husks. In visual assessment, the surface of the millet husk-reinforced composite was noticeably rougher. In many places, filler particles were observed. Single points with holes in the PVC matrix, indicating filler leaching [31], were also noted. In the case of the oat husk-reinforced composite, the filler remained covered in the matrix after exposure. No uncovering particles were observed.

**Figure 6:**
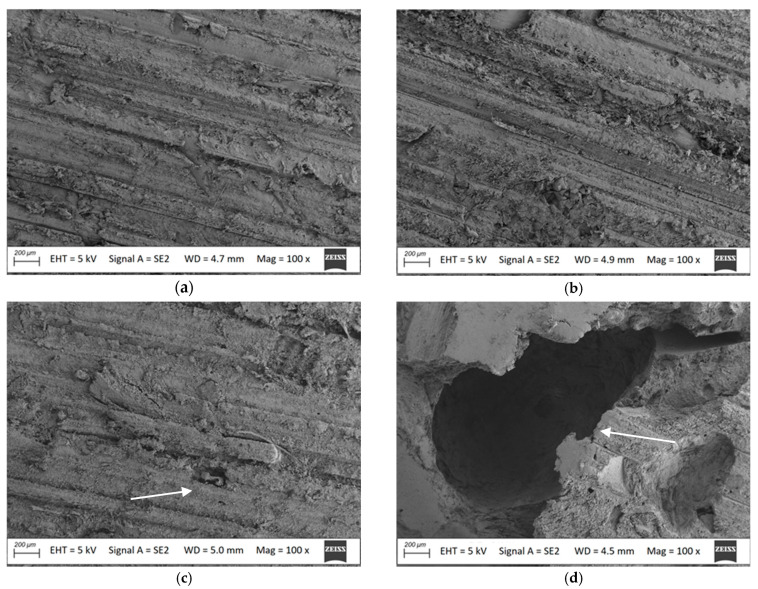

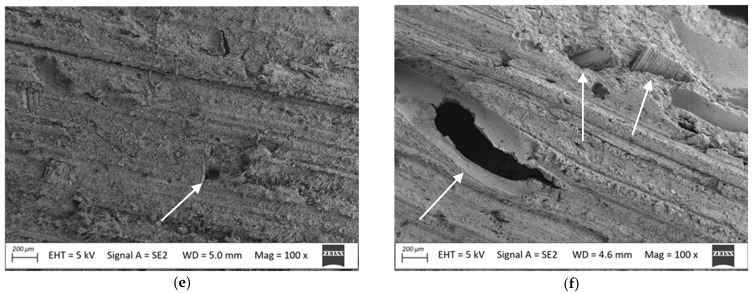
Surface morphology (**a**,**b**) in inital state, oat and millet husk-reinforeced composite, respectively, (**c**,**d**) after mycelium removal with brush, oat and millet husk-reinforeced composite, respectively, (**e**,**f**) after mycelium removal with water jet, oat and millet husk-reinforeced composite, respectively; magnification 100×.

SEM analysis of the cleaned surfaces confirmed the macroscopic observations. The surface of both composites in the initial state had longitudinal grooves, in accordance with the mechanical treatment applied, but without exposing the filler (Figure 6a,b). After exposure to *Coniophora puteana* and removal of the mycelium with the brush in the case of the oat husk-reinforced composite, single, small fragments of filler particles not surrounded by the matrix became visible (Figure 6c). In the case of the millet husk-reinforced composite, large voids in the matrix with smooth surfaces (Figure 6d) were observed after the same cleaning process, indicating that interfacial bonds were lost, resulting in the removal of filler from the polymer matrix [11]. The surface morphology after water jet cleaning was similar (Figure 6e,f), with the millet husk composite showing filler loss of a smaller area than after brush cleaning. It was also revealed that there were non-matrix-covered millet husk particles. The above may indicate that not only the action of *Coniophora puteana*, but also the cleaning technique, is responsible for the filler loss.

### 3.2. Flexural Properties

**Figure 7:**
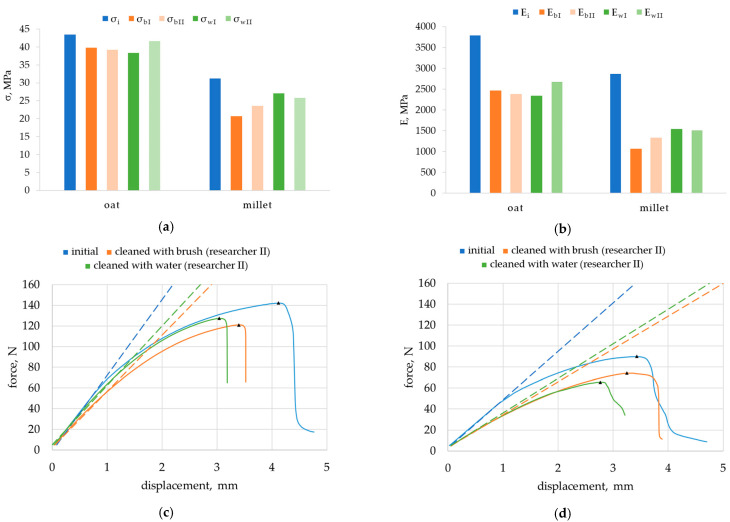
Test results of (**a**) flexural strength σ; (**b**) modulus of elasticity E of oat husk-reinforced composite and millet husk-reinforced composite in initial state (σ_i_, E_i_), after exposure to *Coniophora puteana* and cleaning with brush performed by researcher I (σ_bI_, E_bI_) and by researcher II (σ_bII_, E_bII_) or with water jet, also by researcher I (σ_wI_, E_wI_) and by researcher II (σ_wII_, E_bII_); (**c**) flexural test curves (examples) for oat husk-reinforced composite; and (**d**) flexural test curves (examples) for millet husk-reinforced composite; 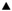 maximum force, the dotted line shows the tangent.

In the initial state, the oat husk-reinforced composite had a flexural strength σ_i_ close to 44 MPa, whereas the millet husk-reinforced composite had it slightly greater than 30 MPa (Figure 7a). The modulus of elasticity E_i_ reached approximately 3800 MPa and 2900 MPa, respectively (Figure 7b).

The analysis of the dispersion of the results and their impact on the significance of the differences in mean results are presented in detail in Section 3.3, therefore, error bars are not shown.

The above differences between oat and millet husk-based product may be due to differences in the shape of the filler [11,25]. The mechanical parameters of the composite depend, among other things, on the quantity, size, and shape of the filler and the degree of its dispersion in the matrix [11,14]. The oat husk particles were predominantly in the shape of long, slender fibres (Figure 1a), favourable in terms of bending and tensile properties [17]. Millet husk particles were plate-like shaped (Figure 1b), which makes proper dispersion in the matrix difficult [11]. Flexural properties are similar to those obtained for composites with rice husk filler [14], although lower than those reported for composites reinforced with sisal [37] or hemp fibres [11,12].

The flexural properties of composites decreased after exposure to *Coniophora puteana*. The flexural strength (the average value of σ_bI_, σ_bII_, σ_wI_, and σ_wII_) of the oat husk-reinforced composite reduced by 8% (Figure 7a); whereas, that of the millet husk-reinforced composite reduced by 10% (Figure 7b). The modulus of elasticity decreased by approximately 50% and 35%, respectively. These changes are illustrated by the flexural curves shown for selected specimens in Figure 7c,d. After exposure, lower values of the maximum force were noted. A flattening of the flexural curve and thus a change in the tangent angle were also observed, especially for the millet husk-reinforced composite.

After exposure and mycelium removal, fragments of filler not surrounded by the matrix were noted (Figure 6c,f) as well as voids in the matrix (Figure 6d–f). This indicates that the interaction between the lignocellulosic particles and the PVC matrix was weakened after exposure [35]. This may be related to the profile surface treatment used. Although SEM images (Figure 6a,b) did not show exposed filler in initial state, as previous studies show, mechanical surface treatment reduces the protection of hydrophilic lignocellulosic particles by the matrix [38]. Even a partially exposed filler is more susceptible to swelling, which leads to a decrease in interfacial adhesion [11,12]. The reduction in the interphase interaction on the composite surface can determine the modulus of elasticity much more than the flexural strength. Weakening of the top layer significantly increases the susceptibility to strain [17]. However, it should be noted that recorded changes are comparable to observed for lignocellulose fibre-reinforced polymer composites exposed to fungi and moisture [19,20,21,26]. The statistical significance of flexural property changes is shown in Section 3.3.2.

### 3.3. Statistical Analyses

#### 3.3.1. Metrological Properties of the Test Method

For all series of results, the null hypothesis for the Shapiro–Wilk test of normality of distribution was confirmed, which means that it can be assumed that the populations from which the result sets described in Table 2 and point 2.4 have a normal distribution.

Grubbs tests showed no single outliers; therefore, all results obtained were used in further analysis.

First, the standard deviation and coefficients of variation of repeatability and reproducibility were estimated for all series of results. Figure 5 shows the results obtained.

**Figure 8:**
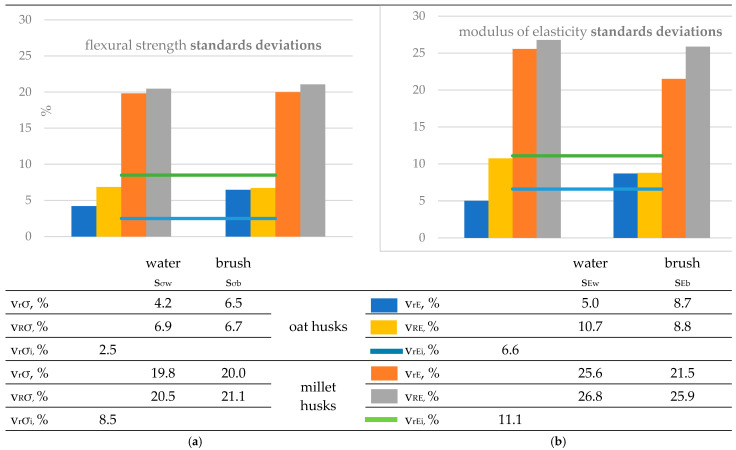
Values of repeatability and within-laboratory reproducibility coefficients of variation for millet husk-reinforced PVC composite and oat husk-reinforced PVC composite samples. v_r_- repeatability coefficient of variation, v_R_—reproducibility coefficients of variation, v_r_σ_i_, v_rEi_ repeatability coefficients of variation values for initial flexural strength and initial modulus of elasticity, respectively, and v_Yw_, v_Yb_-, values of coefficients of variation (v_r_ and v_R_) for the Y quantity after exposure to fungi and cleaning with water jet and soft brush, respectively. (**a**) Flexural strength results and (**b**) modulus of elasticity results.

The repeatability and reproducibility results are presented in Figure 8 in comparison with the repeatability coefficients of variation for the results of the initial flexural properties σ_i_ and E_i_. The repeatability and reproducibility coefficients of variation in the flexural strength and modulus results of both composites after exposure to *Coniophora puteana* and after cleaning were in most cases several times higher than the coefficients in the initial tests. The statistical significance of differences in variances before and after exposure was confirmed using the F-test for the strength variance for the oat composite after cleaning with brush, for the millet composite after cleaning with water and with brush, and for the modulus variance for the millet composite.

Considering that exposure and cleaning can introduce additional sources of variability in the results beyond those obtained from flexural testing alone, larger coefficients of variation seem obvious.

The relative expanded uncertainty of the test method assuming a 95% coverage interval and a normal distribution can be approximately estimated as 2v_R_ and can reach 40–50% of the result value, which poses a high risk when evaluating the product. Considering the possibility that the coefficients of variation in reproducibility will be higher in interlaboratory tests than in intra-laboratory tests, it must be assumed that this uncertainty can be even larger.

It is also evident that the variability in the results expressed by the values of the coefficients of variation differed between the composites. Already in the initial tests, a more than two-fold difference can be observed in the case of $v_{r\sigma i}$ and almost two-fold in the case of the $v_{rEi}$ The differences deepened after exposure and cleaning and reached a four-fold difference between the composites. Therefore, it can be concluded that the uncertainty of the test method depends on the tested material.

Table 3 presents the results of the coefficient of variation between researchers, which was calculated based on the interlaboratory standard deviation S_L_. Considering that the experiment was conducted under conditions of within-laboratory reproducibility, the S_L_ component of the uncertainty can reach significantly higher values in the case of tests in different laboratories.

#### 3.3.2. Differences Between the Water- and Brush-Cleaned Samples

To assess the statistical significance of the differences between the flexural parameters of the samples subjected to different procedures, a one-way ANOVA analysis was performed. Table 4 shows the differences in the results, which were calculated using Equation (12).

(12) $$100\frac{{\overset{̿}{Y}}_{1}-\bar{Y_{i}}}{\bar{Y_{i}}}$$

where the following are defined:

$\bar{Y_{i}}$—result in initial state (mean strength or mean modulus);

${\overset{̿}{Y}}_{1}$—general mean of results (strength or modulus) after exposure and cleaning, calculated according to Equation (7).

To calculate the difference in results for the water jet-cleaned and soft brush-cleaned samples, Equation (13) was used:

(13) $$100\frac{{\overset{̿}{Y}}_{b}-{\overset{̿}{Y}}_{w}}{{\overset{̿}{Y}}_{w}}$$

where the following are defined:

${\overset{̿}{Y}}_{b}$—general mean of results (strength or modulus) after exposure and cleaning with brush;

${\overset{̿}{Y}}_{w}$—general mean of results (strength or modulus) after exposure and cleaning with water.

Table 4 also shows the F-statistic values obtained from the analysis of variance. In all cases, statistical significance was found for the differences in flexural parameters before and after exposure and cleaning (Table 4). It was also observed that in the case of the oat husk composite, the difference in flexural parameters between the water jet- and soft brush-cleaned samples was not statistically significant. These values were 1.3% for flexural strength and 3.6% for modulus. In contrast, in the case of the millet husk composite, the difference was statistically significant. The loss of flexural strength was 16% greater and the loss of modulus was 21% greater with brushing than with water jet cleaning. Therefore, the method of mycelium removal may have caused significant differences in the test results.

**Table 4:** Differences between values of flexural strength and modulus of elasticity for oat husk composites and millet husk composites before exposure (initial tests), after exposure to *Coniophora puteana*, cleaning with water, and after exposure to *Coniophora puteana* and cleaning with a brush. Statistically significant differences are highlighted in grey. F-statistics values resulting from analysis of variance: F_str_—strength results., F_mod_ is the modulus value, and F_crit_ is the critical value for the ANOVA test.

		Oat Husk-Reinforced Composite		Millet Husk-Reinforced Composite	
		Initial Test Results	Cleaning with Water Jet	Initial Test Results	Cleaning with Water Jet
Cleaning with water jet	Difference between values of flexural strength, %	−8		−14	
	Difference between values of modulus, %	−34		−45	
	F_crit_	4.26		4.26	
	F_str_	12		4.73	
	F _mod_	153		64	
Cleaning with soft brush	Mean difference between values of flexural strength, %	−9	−1.3	−28	−16
	Mean difference between values of modulus, %	−36	−3.6	−57	−21
	F_crit_	4.26	4.10	4.26	4.10
	F_str_	14	0.45	21	7.7
	F _mod_	181	1.85	151	9.4

Given that different laboratories may use different cleaning methods, the standard deviation of the reproducibility of the test method may increase. The estimation of the standard deviation of reproducibility for all results after exposure, regardless of the cleaning method, gives the composite with millet husks the coefficient of variation values of 22% for flexural strength and 28% for modulus of elasticity. Assuming a 95% coverage interval and a normal distribution, the expanded uncertainty value of the results can reach 44% for the flexural strength and 56% for the modulus of elasticity. These values may be even higher because the results of this study were obtained based on intra-laboratory reproducibility. When the study is performed by different laboratories, additional factors may also increase the coefficients of variation. This raises the question of whether a test method with such low precision can be used for the assessment of construction products.

## 4. Conclusions

The test procedure for the fungal resistance of construction profiles made of natural fibre composites was analysed in terms of its precision and the influence of a factor not considered in the method model, namely the procedure for cleaning samples after exposure to fungi.

The results showed that the precision of the method is relatively poor. The coefficient of variation related to intra-laboratory reproducibility can take values above 20%, which can lead to uncertainty values of the results of the order of 50% and higher if interlaboratory reproducibility is considered.

It has been shown that different mycelium removal methods can produce different results in resistance tests. Composites with different properties and microstructures may show different susceptibility to changes in the method of mycelium removal.

Considering the above findings, it would be necessary to clarify the test method with respect to the mycelium removal step and other aspects as well.

The issue of the suitability of test methods for assessing the performance of construction products is extremely important and research into the precision and uncertainty associated with testing should be carried out in multiple directions.

Considering the dynamic development of natural fibre composites and their growing significance in civil engineering, further studies are planned. Future studies should cover other biotic factors, including algae and composites, with other fillers.

## Figures and Tables

**Table 1:** The composition of cereal husk-reinforced PVC composites.

Series Name	Matrix	Mineral Filler	Cereal Husk Filler	Other
M	PVC (100 phr)	CaCO_3_ (50 phr)	pulverised millet husks (30 phr)	impact modifiers (4.5 phr) flow modifiers (1 phr) stabiliser (4.5 phr) wax (2 phr)
O			pulverised oat husks (30 phr)	

**Table 2:** Test results series.

Properties	State of samples				
	Initial	After exposure to fungi			
		mycelium removal method			
		soft brush		water jet	
		researcher			
		I	II	I	II
Flexural strength	σ_i_	σ_bI_	σ_bII_	σ_wI_	σ_wII_
Modulus of elasticity	E_i_	E_bI_	E_bII_	E_wI_	E_wII_

**Table 3:** Results of between-researchers coefficient of variation.

Composite	Water Jet Cleaning	Soft Brush Cleaning	Water Jet Cleaning	Soft Brush Cleaning
	$v_{L\sigma w}$, %	$v_{L\sigma b}$, %	$v_{LEw}$, %	$v_{LEb}$, %
Oat husk-reinforced	5.4	1.8	9.5	1.2
Millet husk-reinforced	5.1	6.6	8.0	14.4

## Data Availability

The original contributions presented in this study are included in the article. Further inquiries can be directed to the corresponding author.
